# Improving vaccination rates in older adults and at-risk groups: focus on pertussis

**DOI:** 10.1007/s40520-021-02018-3

**Published:** 2022-01-10

**Authors:** Jung-Hyun Choi, Jaime Correia de Sousa, Monica Fletcher, Giovanni Gabutti, Lauriane Harrington, Michael Holden, Hyungwoo Kim, Jean-Pierre Michel, Piyali Mukherjee, Terry Nolan, Tobias Welte, Stefania Maggi

**Affiliations:** 1 Catholic University of Eunpyeuong St. Mary’s Hospital, Seoul, South Korea; 2 Life and Health Sciences Research Institute (ICVS), School of Medicine, University of Minho, Braga, Portugal; 3 ICVS/3B’s, PT Government Associate Laboratory, Braga, Guimarães Portugal; 4 Usher Institute, University of Edinburgh, Edinburgh, UK; 5 Department of Medical Sciences, University of Ferrara, Ferrara, Italy; 6 GSK, Wavre, Belgium; 7 MNH Associates Ltd, Fleet, UK; 8 Department of Geriatrics, University of Geneva, Geneva, Switzerland; 9 Peter Doherty Institute for Infection and Immunity, University of Melbourne, Melbourne, Australia; 10 Department of Pulmonary and Infectious Diseases, Hannover University School of Medicine, Hannover, Germany; 11 German Center for Lung Research, Hannover, Germany; 12 CNR Aging Branch, Institute of Neuroscience, Padua, Italy

**Keywords:** Pertussis, Vaccination, Tdap, Adults, Elderly, Risk group

## Abstract

Despite the implementation of effective paediatric vaccination programmes, pertussis remains a global health problem. Disease epidemiology has changed over time, shifting towards the adolescent and adult populations. In adults, the true burden of pertussis is greatly underestimated and pertussis vaccine coverage rates are suboptimal, including individuals with chronic conditions. Here, we report the outcomes of a virtual international scientific workshop to assess the evidence on the burden of pertussis in older adults and identify potential solutions to improve uptake of pertussis vaccines. In adults, pertussis is underdiagnosed in part due to atypical or milder clinical presentation and the lack of testing and case confirmation. However, contemporary epidemiological data denoted an increase in the burden of pertussis among adolescents and adults. This might be related to a variety of reasons including the waning of immunity over time, the lack of booster vaccination, and the improved diagnostic methods that led to increased recognition of the disease in adults. Pertussis sequelae can be severe in older adults, particularly those with existing chronic medical conditions, and the vulnerability of these groups is further enhanced by low pertussis vaccine coverage. Possible measures to increase vaccine uptake include strengthening and harmonisation of immunisation guidelines, healthcare professionals taking a more active role in recommending pertussis vaccination, involvement of vaccination centres and pharmacies in the vaccination process, and improving knowledge of pertussis burden and vaccine efficacy among the general population.

## Introduction

Pertussis is an infectious disease of the respiratory tract caused by the bacterium *Bordetella pertussis* [1]. Universal immunisation of the paediatric population has led to a significant decrease in disease-related incidence and mortality in infants [2, 3]. A resurgence of the disease has been observed in the last decade among adolescents and adults [4]. The World Health Organization (WHO) reported a total of 151,074 cases for 2018 across all ages, along with a vaccination coverage of 86% in children [5]. In its annual epidemiological report, the European Centre for Disease Prevention and Control reported 35,627 pertussis cases for 2018, at an average incidence of 8.2 cases per 100,000 population. Sixty-two percent of cases were recorded in individuals ≥ 15 years of age [6]. Available evidence suggest that the burden of pertussis among older adults is greatly underestimated and underdiagnosed, and the real incidence of pertussis may be much higher than is reported [7, 8].

Adolescents and adults can be a source of transmission to infants who are too young to be fully vaccinated against pertussis and who are most susceptible to develop severe disease when infected. Previous studies have shown that household members, especially mothers, are the most common source of pertussis infection in infants less than 6 months old [9, 10]. Maternal immunisation has a key role and is highly effective for prevention of pertussis in newborns and young infants [11, 12]. Healthcare workers who are not protected against pertussis are at high risk for *B. pertussis* exposure and infection and can also transmit the disease to susceptible patients [13, 14].

Adult vaccination strategies against pertussis might be the most effective measure to not only protect the individuals from the disease and its complications, but also mount an indirect protection of new-born infants through immunisation of parents, older siblings, and healthcare workers [9, 15].

Combination vaccines containing reduced-antigen-content tetanus toxoid, diphtheria toxoid, and acellular pertussis (Tdap) are currently widely available for vaccination of children, adolescents and adults [16]. The safety and immunogenicity of Tdap vaccines has been confirmed by numerous clinical trials [17–21], and decennial booster vaccination programmes are now recommended in several countries [22, 23].

The coronavirus disease (COVID-19) pandemic has highlighted the value of vaccination of vulnerable adult populations as a preventive measure against infectious diseases. The International Council on Adult Immunization stressed the urgent need for global adult immunisation strategy that covers available vaccines against a variety of pathogens other than the severe acute respiratory syndrome coronavirus 2 (SARS-CoV-2) and called upon global community and various stakeholders to implement evidence-based adult immunisation programmes and policies [24].

**Fig. 1:**
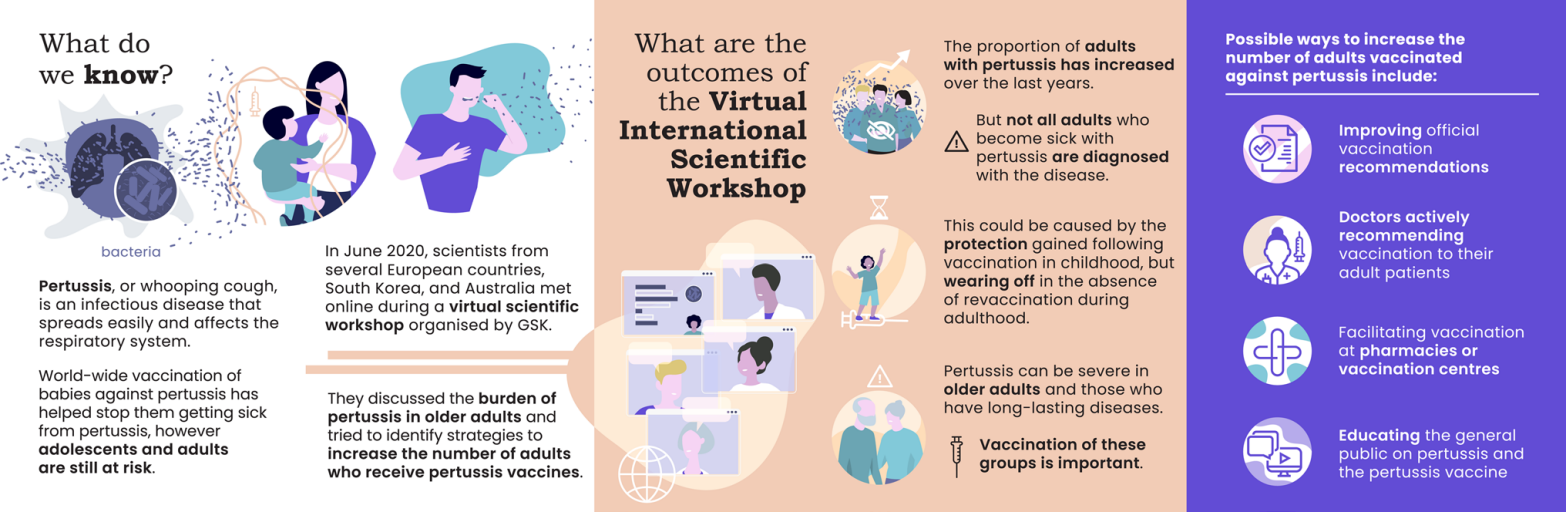
Plain language summary

The authors, stakeholders, and experts in the field of adult vaccination from multiple countries (Australia, Belgium, Germany, Italy, Portugal, South Korea, Switzerland, and United Kingdom) gathered during a virtual scientific workshop organised by GSK on the 30th of June 2020. The objectives were to (1) assess the evidence on the burden of pertussis in older adults and those with respiratory conditions and (2) identify solutions to improve uptake of Tdap vaccines. This manuscript summarises those discussions. A lay language graphical summary is also available in Fig. 1.

## Pertussis epidemiology: not just a childhood disease

Adults > 18 years of age are now proportionally more affected by pertussis than 10–20 years earlier [7, 8, 25]. In Sweden, 59% of the total laboratory-confirmed pertussis cases were reported for adults > 18 years old in 2018 as compared with 4% of cases reported in 1998. Among older adults > 50 years of age, these rates were 17.3% (2018) compared with 1.3% (1998) [26]. Similar trends have been observed in Germany [27] and Australia [28]. This resurgence can be attributed to several factors such as low vaccine coverage, waning immunity, and lack of booster vaccination, but also improving diagnostic methods and increasing recognition of the disease in adults [3, 29].

Immune responses against pertussis, whether natural or acquired through vaccination, decline over time [30]. Booster vaccinations to maintain high antibody levels are needed for the prevention of the disease [3]. Although slight fluctuations of the worldwide vaccination coverage in children have occurred, it has been estimated at 81.6% in 2019 [31]; however, vaccination coverage among adults is low. It has been estimated that about 4.1 million Australians are not vaccinated under the National Immunisation Program each year [32]. Similarly, Tdap vaccination coverage among adults aged ≥ 19 years in the United States (US) was 31.7% in 2017 [33]. Limited access to vaccines, lack of provider recommendations, low public awareness of pertussis and the benefits of vaccination, misinformation, and fear of vaccines’ side effects are potential reasons behind this low coverage. From the healthcare practitioners’ (HCPs) perspective, patients forgetting about their appointments, inadequate access to vaccination and language barriers, are perceived as major barriers for vaccine acceptance [34].

## Burden of pertussis in adults

Today, the demographic dispersion of pertussis includes adolescents and adults, in addition to infants. Furthermore, the true burden of pertussis in adolescents and adults is greatly underestimated [8, 35].

One factor of underestimation is related to the atypical clinical characteristics of cases compared to the classical child symptoms, making the diagnosis difficult [36]. After the incubation period, the typical pertussis infection course starts with a catarrhal stage, during which symptoms closely resemble a mild upper respiratory tract infection, followed by a paroxysmal stage and convalescent stage [1, 37]. During the paroxysmal stage, children experience intense and violent coughing that last several minutes and is accompanied with a whooping sound when breathing in. Although adolescents and adults present similar symptoms, these might be milder than in children, resulting in failure to diagnose or misdiagnosis. According to a recent systematic review, paroxysmal cough, inspiratory whooping and post-tussive vomiting are the three most common clinical features of pertussis in adults that can be considered by physicians for the clinical diagnosis of the disease [38]. In a study of 27 adolescents and adults, all individuals had paroxysmal cough, 26% had whooping, and 56% had post-tussive vomiting [39]. The complications of coughing can include urinary incontinence (mainly reported in women), fainting, and rib fractures. Many others clinical features are classically reported as pharyngeal, influenza-like symptoms, sinus pain, sneezing attacks, hoarseness, headaches, and sweating attacks [36, 40].

Besides atypical presentation of pertussis in older adults, late presentation to HCPs (leading to no or late testing) contribute to under-diagnosis of the disease in this age group. Given the limited sensitivity of laboratory diagnostic methods, there is a need for more and earlier testing of patients presenting with a cough. Awareness of the symptoms of pertussis (including atypical ones) in adults and of the time-sensitivity of laboratory testing needs to increase.

Another cause of underestimation is the challenge associated with laboratory case confirmation. Culture of nasopharyngeal secretions and polymerase chain reaction are traditionally used for diagnosis but are effective only at the early phase of the disease. Patients, however, may present late to HCPs and it typically takes several visits to reach a diagnosis. Serologic testing targets pertussis toxin, but results can be affected by pre-existing immunity (induced either by previous vaccination or infection). Moreover, serologic methods for the diagnosis of pertussis are not standardised [41].

An additional concern of geriatricians is the possible links of *B. pertussis* with dementia. This is based on the well-established correlation existing between systemic inflammation and Alzheimer disease (AD) and accounts one intriguing epidemiologic observation [42]. The subclinical nasopharyngeal *B. pertussis* colonisation, in close proximity to the central nervous system and olfactory pathway, could explain how *B. pertussis* and its toxin account for the activation of the microglia, inflammation, atrophy, and accumulation of the amyloid plaques and tau tangles in the brain [43]. In addition, vaccination against pertussis has been associated with a significantly reduced risk for AD [43]. While this new hypothesis of the potential involvement of *B. pertussis* in the aetiology of AD is still the topic of ongoing debates, in a life-course perspective, the important role of infant vaccinations and boosters later in life is well-established.

During the workshop, extensive discussions were conducted on the long-term consequences of pertussis and the increased impact from comorbidities. The clinical course and the potential complications and sequelae of the disease may also be severe among older adults, not just in infants. Studies suggest that older adults have an increased risk for pertussis-related mortality than children or adolescents, though it is likely to be underestimated [8]. Complications, such as apnoea, pneumonia, sinusitis, and otitis media also increases with age [8, 44]. Other complications are less frequent and include seizures and encephalopathy (cerebral hypoxia related to asphyxia), subarachnoid and intraventricular haemorrhages, and tetanic seizures [45]. Individuals with underlying conditions, such as asthma, chronic obstructive pulmonary disease (COPD), and obesity are at an even higher risk to develop a severe form of the disease [46–48], and recent studies suggest that pertussis incidence in COPD populations may be grossly underestimated [49, 50]. Pertussis has been associated with an increase in healthcare resource use in patients with COPD and asthma, increasing the overall economic burden, exacerbation, and hospitalisation rate related to the disease [51–53].

The COVID-19 pandemic has highlighted the vulnerability of older adults to respiratory infectious diseases and their complications. It has been revealed that individuals with comorbidities are more vulnerable to infection and are more prone to develop severe symptoms. Some of the most common comorbidities in hospitalised COVID-19 patients were diabetes and obesity [54, 55], conditions also associated with an increased risk of pertussis infection in adults.

## Immunisation strategies for adults

Vaccination is one of the most effective strategies to prevent infection and reduce severity of pertussis in adolescents and adults [1]. Antibodies against *B. pertussis* antigens play a fundamental role in the protection against pertussis. There is, however, no correlate of protection, meaning that antibody levels cannot be precisely correlated with clinical protection [56]. Tdap vaccines have been demonstrated to be well tolerated and highly immunogenic in adults [57]. Although immune responses against pertussis antigens decline over time, they remain several fold higher than pre-vaccination levels for 10 years after booster vaccination. The decennial booster dose mounts a strong anamnestic response for all tested pertussis antigens. Although injection site reactions are common, the vaccine is generally well tolerated [18, 21, 30, 58].

Since 2005, the US Advisory Committee on Immunization Practices recommended vaccination with one dose of Tdap for previously unvaccinated adolescents aged 11–18 years and adults aged 19–64 years. This recommendation was expanded to adults aged > 65 years in 2012 [59]. The 2011 updates of the recommendation also included the immunisation of unvaccinated pregnant women and all household contacts of newborns who had not been vaccinated [60]. Since 2019, adolescents aged > 11 years and adults who have never been vaccinated with Tdap before, should receive one dose as soon as possible, followed by another two doses (Td or Tdap) at 4 weeks and 6–12 months after the first dose. Thereafter, a booster dose should be administered every 10 years [61]. Healthcare workers in close contact with patients should also receive a Tdap dose [62]. In the United Kingdom, routine immunisation against pertussis does not include adolescents ≥ 10 years of age and adults, except for pregnant women and during outbreaks [63]. However, a booster dose is recommended for healthcare personnel working with infants if they did not receive a dose in the preceding 5 years [64]. National policies for the immunisation of healthcare workers against pertussis are in place in 19 other European countries [65]. Maternal immunisation as well as a decennial booster with Tdap is also recommended in several European countries [22].

## Strategies to improve adult vaccination coverage

In contrast with the fairly high vaccination coverage achieved in children, that is currently hampered by the COVID-19 pandemic [66], optimisation of adult vaccination was not successfully accomplished in any country until now, resulting in a worldwide under-immunisation of this age group.

During the COVID-19 pandemic, most national and international agencies, such as the WHO, the Centers for Disease Control (CDC), the Robert Koch Institute, and the Italian public health authority have all reinforced their recommendations on adult vaccination, including Tdap, to help protect HCPs and the residents of care facilities, and to reduce the risk of double infection, the number of possible clinical visits, and the difficulties related to differential diagnosis of COVID-19 and other respiratory infections with similar clinical features [67–70]. This may generate a shift in attitude and awareness of vaccine preventable diseases in both HCPs and the public.

There are multiple barriers found at the patient, HCP, infrastructure, and funding level that prevent reaching a high vaccine uptake among adults. The belief by some HCPs and patients that childhood vaccination provides lifelong protection, the low awareness of pertussis as an adult disease, as well as the low awareness of safety and benefits of adult pertussis vaccines might lead to lack of recommendation among HCPs and to vaccine hesitancy or refusal among patients [34, 71]. Extensive education on the disease and the value of vaccination initiatives would raise awareness of the benefits of adult vaccination. Even when vaccination is recommended, coverage rates in adults are low, highlighting that public health authority recommendation is not sufficient. As shown in different studies, HCP recommendations may increase vaccine uptake in their adult patients [32, 72]. Thus, vaccination needs to be part of the HCP’s care plan for patients and cover all adult vaccines.

Implementation of vaccination programmes for high-risk adults is even more troublesome, likely due to difficulties in identifying and reaching out to these people. Disparities of recommendations in different countries may impede efforts to improve adult vaccination. International guidelines would create a harmonised recommendation for adult vaccination.

The vaccination process involves many stakeholders: general practitioners, nurses, pharmacists, geriatricians, specialists (e.g. pulmonologists for asthma and COPD), and funding bodies. Cooperation across disciplines and societies could improve pertussis awareness and adult vaccination compliance. Vaccination centres other than primary care providers, such as community pharmacies can be an attractive channel and be an efficient way to improve vaccine access and coverage among adults [73, 74]. People visit the pharmacist four times more often than other HCPs, pharmacies are already involved in dispensing vaccines, and some countries have legislation allowing vaccination at the pharmacy [75]. During the 2014/2015 and 2015/2016 influenza seasons, 115,000 doses of influenza vaccine were delivered in pharmacies in Ireland; this represents about 10% of all administered influenza vaccines. During the 2020/21 influenza season, community pharmacies in England delivered 2.6 million doses of influenza vaccine, which is 53% higher compared to the 2019/20 season, and are also supporting the COVID-19 vaccination programme [76]. In countries where pharmacy vaccination was introduced (Canada, the US, Portugal), flu vaccine coverage increased [75].

Waning of immunity, immunosenescence, and burden of pertussis in adults provide a strong rational for booster vaccination, considering immunogenicity and safety of Tdap. It might have better acceptance among older adults than the concomitant administration of Td and monovalent pertussis vaccines. Implementation of Tdap for the decennial booster might also be cost-effective [77]. Randomised clinical trials designed to establish Tdap efficacy in the older adults are still needed, however, the effectiveness of Tdap vaccination has been demonstrated in older adults [78].

Tdap vaccination in the COVID-19 era was also discussed during the workshop. On one hand, despite the value of adult vaccination being reinforced both by the current pandemic situation and by several international and national recommendations, less funding is likely to be directed towards the diagnosis of pertussis and Tdap vaccination in the near future. On the other hand, disease severity and the high morbidity and mortality observed among older adults and at-risk populations during the COVID-19 pandemic may redound to adult vaccination efforts and direct more attention towards protection of adults and at-risk groups. As pertussis is difficult to diagnose and it could increase the risk of hospitalisation in adults [79], vaccination may also have the potential to relieve pressure on healthcare systems in difficult times. The role of pharmacists in immunisation programmes would be even more important while the SARS-CoV-2 is still circulating, though comprehensive training programmes are required for them to be able to administer vaccines where this is not a standard. Finally, there is an increased public awareness of respiratory infectious disease, even among patients, but fear of COVID-19 may drop willingness to attend primary healthcare sites for vaccination.

## Conclusions

Pertussis is still a frequent infectious disease, with high burden among adolescents and adults, particularly for those with underlying health conditions. This adult population represents a significant source of transmission for unvaccinated or partially vaccinated infants. Atypical clinical presentation of pertussis in adolescents and adults makes clinical diagnosis difficult. Despite recent improvements, a more accurate epidemiological surveillance is desirable to assess the real burden of disease and the impact of vaccination. Pertussis control in older adults could be improved by engaging scientific leaders for a multidisciplinary approach of pertussis and its complications, obtaining a political commitment for the need of research and long-term vaccination policies, and by creating a dialogue on the value of vaccination to increase public engagement.

## Data Availability

Not applicable.
